# Using System Dynamics Approach to Explore the Mode Shift between Automated Vehicles, Conventional Vehicles, and Public Transport in Melbourne, Australia

**DOI:** 10.3390/s23177388

**Published:** 2023-08-24

**Authors:** Yilun Chen, Peter Stasinopoulos, Nirajan Shiwakoti, Shah Khalid Khan

**Affiliations:** School of Engineering, RMIT University, Melbourne, VIC 3000, Australia; s3598361@student.rmit.edu.au (Y.C.); peter.stasinopoulos@rmit.edu.au (P.S.); s3680269@student.rmit.edu.au (S.K.K.)

**Keywords:** system dynamics, driverless vehicles, future transportation, transport policy, smart mobility

## Abstract

With the increasing use of automated vehicles (AVs) in the coming decades, government authorities and private companies must leverage their potential disruption to benefit society. Few studies have considered the impact of AVs towards mode shift by considering a range of factors at the city level, especially in Australia. To address this knowledge gap, we developed a system dynamic (SD)-based model to explore the mode shift between conventional vehicles (CVs), AVs, and public transport (PT) by systematically considering a range of factors, such as road network, vehicle cost, public transport supply, and congestion level. By using Melbourne’s Transport Network as a case study, the model simulates the mode shift among AVs, CVs, and PT modes in the transportation system over 50 years, starting from 2018, with the adoption of AVs beginning in 2025. Inputs such as current traffic, road capacity, public perception, and technological advancement of AVs are used to assess the effects of different policy options on the transport systems. The data source used is from the Victorian Integrated Transport Model (VITM), provided by the Department of Transport and Planning, Melbourne, Australia, data from the existing literature, and authors’ assumptions. To our best knowledge, this is the first time using an SD model to investigate the impacts of AVs on mode shift in the Australian context. The findings suggest that AVs will gradually replace CVs as another primary mode of transportation. However, PT will still play a significant role in the transportation system, accounting for 50% of total trips by person after 2058. Cost is the most critical factor affecting AV adoption rates, followed by road network capacity and awareness programs. This study also identifies the need for future research to investigate the induced demand for travel due to the adoption of AVs and the application of equilibrium constraints to the traffic assignment model to increase model accuracy. These findings can be helpful for policymakers and stakeholders to make informed decisions regarding AV adoption policies and strategies.

## 1. Introduction

Automated driving technologies (e.g., artificial intelligence and remote sensing) have received much attention for their research and developments [1]. Automated driving technologies can transfer vehicle driving functions from human drivers to computers, and the automation level is divided into six levels [2]. Simply defined, level 0 means no driving automation, while level 5 demonstrates full driving automation without any human intervention. Moreover, AVs could improve road safety by eradicating traffic accidents, as most accidents are due to human errors, such as driving too fast and driver fatigue. In short, the upcoming automated vehicles will benefit the broader society by decreasing traffic congestion, offering new mobility choices, and reducing road accidents [3].

The rise in AVs is expected to significantly affect the transportation sector by changing the way people travel. AVs have the potential to revolutionise mobility by reducing traffic congestion, improving road safety, and increasing energy efficiency [4]. However, there are concerns that the widespread adoption of AVs could lead to an increase in vehicle kilometres travelled and a decrease in the use of public transport (PT) and active transportation modes, ultimately increasing energy consumption, emissions, and congestion [4]. Therefore, it is important to investigate the potential mode shift between AVs, CVs, and PT to evaluate the effect of AVs on the transportation system and plan accordingly. Most studies employed a static approach to investigate the effect of AVs on the transportation system without considering the dynamic interactions between different travel modes and the feedback loops that could affect the mode shift behaviour [5,6].

If we want to manage future road networks to meet the demands of automated vehicle trips due to the shift from public transport and conventional vehicle trips, we need to understand how AV trips change over time due to a range of reasons, such as policy implementation, AV cost, and psychological factors. As such, the main contributions of this study are as follows:

1. We developed a system dynamic (SD)-based model to explore the mode shift between conventional vehicles (CVs), AVs, and public transport (PT) by systematically considering a range of factors, such as road network, vehicle cost, public transport supply, and congestion level. This model addresses the knowledge gaps on the impact of AVs towards mode shift by considering a range of factors at the city level.
2. Inputs such as current traffic, road capacity, public perception, and technological advancement of AVs are used to assess the effects of different policy options on the transport systems. An SD approach has been adopted for the present study because it can incorporate the dynamic interactions [7] between different travel modes and the feedback loops that could affect the mode shift behaviour. To our best knowledge, this is the first time using an SD model to investigate the impacts of AVs on mode shift in the Australian context.
3. The SD model provides a valuable contribution to the methodological understanding of the effects of AVs on transportation by considering various system-level factors. The model can be used to explore the effects of AV adoption on mode shift, changes in traffic congestion, and other transportation-related factors, supporting policy decision making to achieve a sustainable, equitable, and accessible transport system, especially for the long term. This model also presents significant advantages. The SD model not only comprehensively considers various factors and their quantitative relationships, but it also allows for sensitivity analysis of individual variables. This capability enables us to thoroughly investigate the influences of each variable, enhancing the model’s comprehensiveness and utility. Additionally, the SD model is a powerful tool for analysing the complex interactions between different components of the transportation system and identifying potential solutions to the challenges posed by AV adoption. By providing a detailed analysis of the effects of AV adoption on modal shift behaviour, the proposed model can help policymakers develop policies that promote the adoption of AVs while also minimising the negative effects on PT and congestion.

The paper is organised as follows. Section 2 presents the literature review for the transport modelling and system dynamics approach. It is followed by Section 3 that describes the SD model developed for this study, while Section 4 discusses the results for different scenarios. A list of the abbreviations used in this study is shown in Table 1.

## 2. Literature Review

In this section, we review the relevant works under two subsections: transport modelling and system dynamics modelling.

### 2.1. Transport Modelling

Some past studies investigated the effects of AVs on traffic flow and traffic safety using microscopic traffic simulations of individual vehicles [8,9]. Other interesting studies also investigated the effects of AVs using microscopic traffic modelling. For example, [10] researched lane assignment strategies of AVs and their effects on overall traffic efficiency and safety in a highway scenario. Reference [11] used the traffic simulation package VISSIM to investigate the congestion effects of shared AVs on urban traffic by modelling the peak morning period in 2040. Several shared AV market penetrations were modelled: 0 per cent, 3 per cent, 25 per cent, 50 per cent, and 100 per cent. Similarly, [12] developed an efficient stochastic optimisation framework to find optimal shares between CVs and AVs by considering factors of CAVs (e.g., VKT, the value of time, and automation cost). This framework was successfully applied to the Chicago network, and the system costs were optimised. Reference [13] studied a mixed traffic system to control the density and ratio of CVs and AVs to avoid large-scale traffic congestion using a cellular automation model. It was suggested that the findings would have practical implications for traffic management control. From a traffic safety perspective, mixed traffic flow was simulated to identify the frequency of dangerous situations and the value of time to collision under different penetration levels [14]. The results revealed that smooth driving increases with the CAV penetration rate. Another study conducted a detailed assessment of the effects of CAVs on a freeway using a microsimulation [15]. The findings showed that CAVs could reduce delay and emissions by 38 per cent and 52 per cent in shared lanes. Shared lanes performed better at low traffic volumes, while dedicated lanes performed at high volumes. A recent study by [16] used SDs to optimise mobility by understanding mode choice between rail, car, bus, and air. In addition, the SD method was implemented in EV adoption by incorporating cost, infrastructure supply, vehicle technology, and social utility [17]. In policy developments, various factors (e.g., GDP, capital investment, and solid waste emission) were modelled in SDs to evaluate policy effects on the urban economy [18].

Further, a study by [19] proposed a multi-stage modelling approach to enhance network performance to cater to the growing demand for AVs. While the AV-related subnetwork could improve network performance, it also increased the total travel distance. In a recent study, [20] proposed a new business model for AVs called ‘AV crowdsourcing’, which involved renting out privately owned AVs to gain profits. The study tested the feasibility of this business scenario using an equilibrium model. However, the optimal price for AV crowdsourcing needs to be investigated further by considering individuals’ sensitivity to the utility function and additional costs associated with AVs. This business model has the potential to provide a high return for private AV users. However, more research is needed to fully understand its effect on the adoption rate of AVs and the overall transportation system. Reference [21] predicted that urban households would see a 2.8 per cent increase in commuting trips using private AVs by coupling North Carolina’s demand and choice models to capture household preference. However, the result varied by different penetration rates and fuel types.

In summary, most past studies explored the effects of AVs on traffic flow, efficiency, and safety using microsimulations. Given that Australia will establish an AV safety law in 2026 to facilitate the deployment of AVs [22], this suggests a need for further research into the external factors affecting future AV trips. However, few studies have considered the effect of AVs on mode shift by considering a range of factors at the system level.

### 2.2. System Dynamics Modelling

This literature review primarily focuses on the application of SD modelling to transport planning, especially the effects of AVs and EVs. Reference [23] examined the possible implications of implementing AVs by employing an SD approach to three scenarios: (1) no change in behaviour and ownership, (2) change in behaviour and no change in ownership, and (3) complete change in ownership in which all vehicles are shared AVs. However, the investigation did not consider the adoption process, including factors such as penetration level and level of service, which may change over time. Additionally, the data were gathered through a workshop setting. In addition, while a study conducted in the Netherlands used an SD modelling approach to examine the adoption process and policy tests across four scenarios (i.e., AV in bloom, demand, doubt, and standby), it failed to account for the potential traffic congestion resulting from AV usage and the associated policy effects, such as congestion charging policies [24]. A comparable study by [25] employed an SD modelling approach to assess the effects of AVs on mode choice, focusing on levels 1 to 3. The study analysed two scenarios: AVs and cooperative/connected vehicles, which can communicate with infrastructure and other vehicles. However, the base year data used in the study were from 2013, which may not accurately reflect the current state of AV technology, as it has been rapidly advancing in recent years. Some past research leveraging SD modelling to solve complex interactive transport problems is shown in Table 2.

As shown in Table 2, in previous studies, SD modelling has been used to explore various facets of AV adoption and its influence on transport planning. However, few studies considered factors, such as network capacity and current transport characteristics, to evaluate the adoption of AVs at the system level (i.e., city level). As AV technology is continuously evolving and maturing, it is crucial to conduct further research into adoption rates of AVs compared with trips from other modes, such as PT and CVs, in a city-level context. Currently, there is a lack of a comprehensive framework to systematically consider mode shift change due to the upcoming AVs, especially in an Australian context.

## 3. Methods

The SD model in this study considers the interaction of AV and CV adoption in a mixed-vehicle fleet along with PT. It is developed using VENSIM PLE (version 8.2.0) and simulated from 2018 to 2068, with AV adoption starting in 2025 [28]. The model dynamically computes parameters through feedback loops to determine their impact. System dynamics modelling involves the creation of stock-and-flow models, where flows are divided into inflows and outflows, representing the rates at which quantities are added or subtracted from a specific stock. Consequently, the integral of the net flow, combined with the initial stock value at time “a0”, yields the total stock at time “a”. The net flow, calculated by subtracting outflows from inflows, represents the derivative of the total stock concerning time, as shown in Equation (1).

(1) $$Stock\;\left(a\right)=\;\int_{a0}^{a}\left[inflow\left(a\right)-outflow\left(a\right)\right]da+Stock(a0)$$

### 3.1. Description of the System in This Study

**Figure 1:**
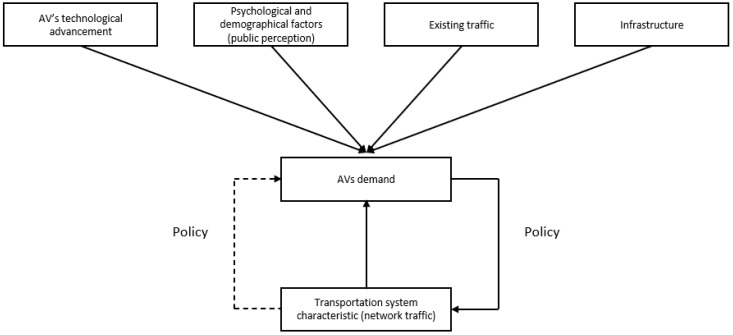
System effects in this study.

The system under consideration is described in Figure 1. External factors are important in determining future AV demand, such as technological advancement and infrastructure capacity of AVs. In this study, AVs represent level 2 and above. Different policies can affect an individual’s mode of choice between PT, AVs, and CVs. In addition, if more individuals use CVs or AVs instead of PT, the results could prevent them from using private vehicles because of increased travel time. Thus, the government will adjust the policy (dotted line) once more network traffic is causing congestion. Therefore, policy decision making is important to achieve a sustainable, equitable, and accessible transport system by satisfying equilibrium in the system.

### 3.2. Model Explanation

#### 3.2.1. Data Input

We obtained the transport data from VITM developed by the Victorian Department of Transport and Planning (DTP), Melbourne, Australia. VITM is a strategic four-step transport demand model developed to predict future travel demand and travel patterns as a result of land use changes, population changes, travel behaviour changes, and major infrastructure projects [29]. VITM is based on the Victorian Integrated Survey of Travel and Activity, including individual trips within family households. The model incorporates the complicated interactions within the transport system (e.g., private vehicle trips, PT, and other modes) and land use changes. Specifically, the VITM is a comprehensive transport demand model that operates on multiple time periods, trip purposes, and modes of travel. This model encompasses car, public transport, and active transport modes and is designed to estimate transportation demand over a typical school day. Employing population, employment, and enrolment projections, VITM assesses the forthcoming impacts of changes in Victoria’s road and public transport infrastructure. Further, we considered data from the existing literature [30,31] and the national survey conducted in Australia [32], and where the relevant data were unavailable, we made realistic assumptions, which are shown in the following tables.

#### 3.2.2. Calculations

**Figure 2:**
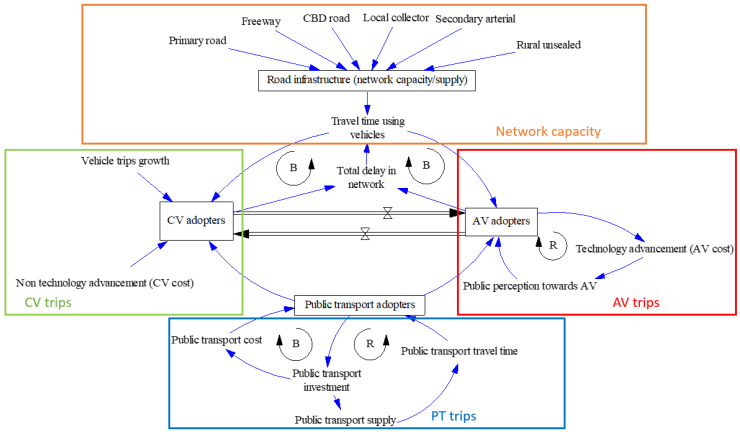
Simplified architecture of the stock-and-flow model.

**Figure 3:**
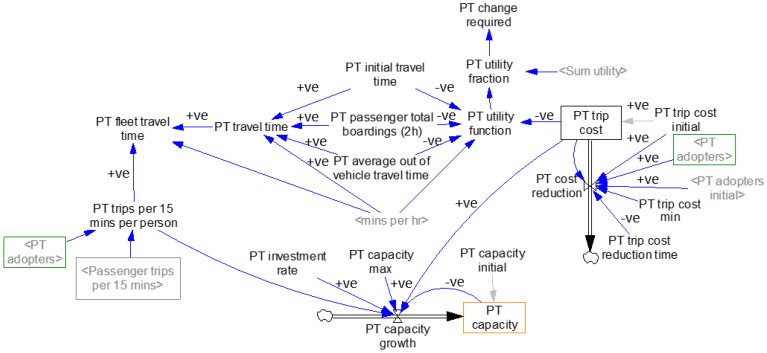
Public transport adoption sub-model. (Notes: 1. <> symbols signify their repeated occurrence within the system dynamics model, whereas variables lacking <> symbols appear only one time in the system. 2. <min per h> denotes a conversion factor of 60 min per h, facilitating unit conversion within this model).

**Figure 4:**
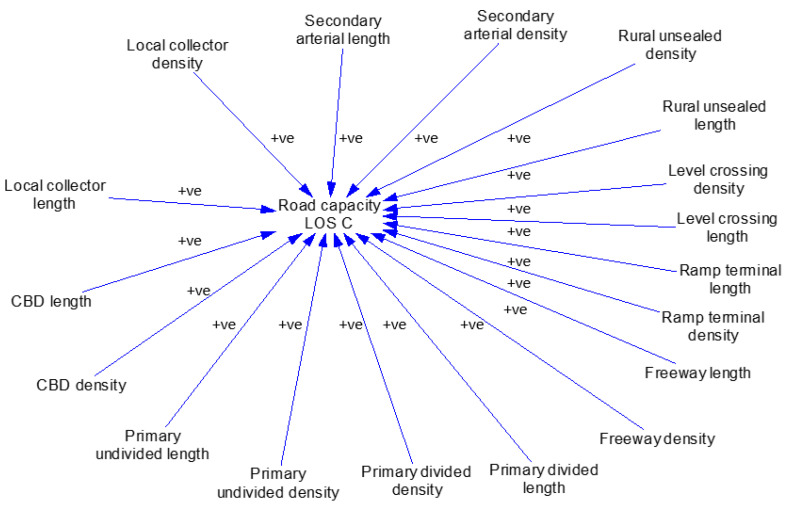
Network capacity sub-model.

**Figure 5:**
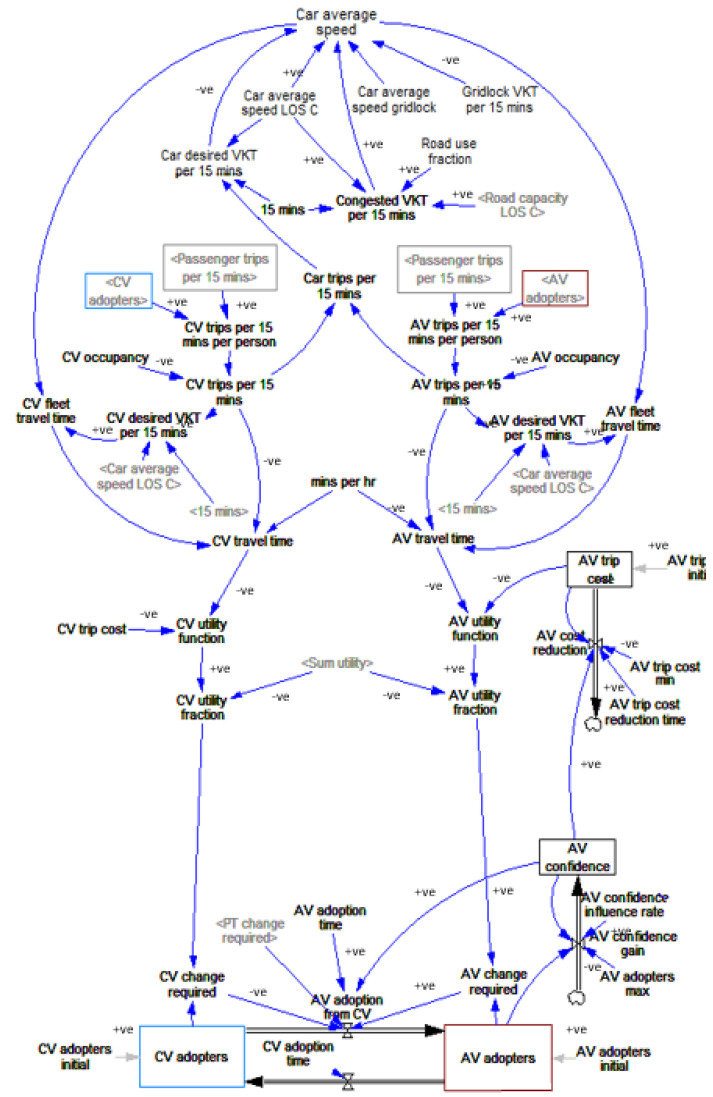
Transitions between CV and AV sub-model. (Notes: 1. <> symbols signify their repeated occurrence within the system dynamics model, whereas variables lacking <> symbols appear only one time in the system. 2. ‘AV trip cost’ is represented as a stock due to its dependency on other variables like ‘AV trip cost initial’ and ‘AV cost reduction’. In contrast, ‘CV trip cost’ maintains a more consistent cost due to its mature technology. Consequently, ‘AV trip cost’ is categorised as a stock, while ‘CV trip cost’ is a variable unaffected by other factors).

The study proposed a stock-and-flow-based SD model to simulate the distribution of trips among CVs, AVs, and PT. The model incorporates four sub-models: network capacity, CV trips, AV trips, and PT trips, as shown in Figure 2. As shown in Figure 3, Figure 4 and Figure 5, the stocks (e.g., CV adopters) are represented by boxes, while the double-lined arrows represent flows (e.g., the total delay in the network). The ‘tap’ symbol denotes flow rates (e.g., from CV adopters to AV adopters), and the single-lined arrows represent influence links (e.g., local collectors influencing road infrastructure). An encircled R represents the reinforcing feedback loop, while an encircled B represents a balancing feedback loop. The model takes inputs and time series data to generate outputs. The simulation model was used to explore the effects of AV adoption on the transportation system at a city level, including the shifts in mode share and changes in traffic congestion. The model outputs include CV adopters, AV adopters, and PT adopters, representing any 15 min period during a typical weekday AM (7 am to 9 am) peak, signifying that the system dynamics model simulates 15 min segments within the AM peak of a standard weekday. Consequently, we have performed a straightforward calculation to derive 15 min boardings, achieved by dividing the 2 h duration by 8. The simulation period is 50 years, starting from the base year.

### 3.3. Sub-Model Explanation

The sub-model of the study includes public transport, network capacity, and CV transitions to AV, which are used to evaluate the various factors influencing AV adoption in a closed transportation system.

#### 3.3.1. Public Transport Sub-Model

Figure 3 shows the public transport sub-model. The trips generated by public transport depend on the adoption by AV and CV users. Table 3 shows the sub-model’s equations, values, and units for the key variables and stocks.

The utility function determines the number of people who choose PT as their primary mode of transportation [33]. The choice of PT as a primary mode of transportation is influenced by various factors, including travel time, travel cost, and standard deviation of travel time. In this study, VITM was used to determine the total PT travel time, which was used to obtain the average PT travel time by dividing the total PT boarding. The average PT travel time also included the average out-of-vehicle travel time, such as the time spent walking to the tram/train station. For example, the “PT utility function” hinges on two pivotal factors: the “PT trip cost” and “PT travel time”. The “PT trip cost” element can be influenced by the uptake of PT by individuals, denoted by “PT adopters” and “PT adopters initial”, thereby influencing what we term as “PT cost reduction”. Furthermore, the “PT travel time” is sourced from the VITM model’s 2018 dataset. As more individuals embrace PT, it might drive the “PT investment rate”, thereby impacting “PT capacity growth” and, subsequently, the number of “PT adopters”. Additionally, ‘PT utility function’ refers to a mathematical construct employed for assessing passenger modal preferences, while ‘PT utility fraction’ utilises this function to ascertain the likelihood of selecting the PT mode over AV and CV modes.

**Table 3:** The public transportation sub-model’s parameters, equations, and values.

Parameter Name	Unit	Value (Equation)	Source/Explanation
Sum utility	N/A	EXP (CV utility) + EXP (AV utility) + EXP (PT utility)	Utility function [34].
PT change required	N/A		Percentage changes in individuals opting for public transport as their primary mode during each simulation interval.
PT utility function	N/A	−0.049 × (PT initial travel time × min per h/”PT passenger total boardings (2 h)”) − 0.05 × PT average out-of-vehicle travel time − 0.0038 × PT trip cost	Probability of choosing PT as commuting mode based on travel time and cost during any 15 min at AM peak.
PT initial travel time	Person × hour	165,795	Collective travel duration via various modes such as trains, trams, and buses, as supplied by the VITM model from DTP for input into this system dynamics model.
PT passenger total boarding (2 h)	Person	508,420	Cumulative count of person boardings on public transport encompassing train, bus, and tram trips. This information is furnished by the VITM model from DTP during the AM peak period spanning 2 h.
PT average out-of-vehicle travel time	Minute	11	VISTA provided by DTP.
PT travel time	Minute	PT initial travel time × min per h/“PT passenger total boardings (2 h)” + PT average out-of-vehicle travel time	PT travel time includes in-vehicle travel time and out-of-vehicle travel time.
PT fleet travel time	Person × hour	PT trips per 15 min per person × PT travel time/min per h	Total public transport fleet travel time including trains, trams, and buses.
PT trips per 15 min per person	Person	Passenger trips per 15 min × PT adopters	It is to determine the number of people who choose PT modes across total people.
PT investment rate	Dmnl/Year/Person/dollar	1 × 10^−9^	Amount by which ‘PT capacity’ grows each year for each dollar spent on PT.
PT capacity max	Dmnl	0.5	Fraction of passenger travel that PT can ultimately service.
PT capacity growth	Dmnl/Year	PT trip cost × PT trips per 15 min per person × PT investment rate × (PT capacity max − PT capacity)/PT capacity max	

The adoption of PT by users can contribute to PT-related revenue, which can then be used to increase PT capacity by providing more services. The PT investment rate is the amount by which PT capacity grows each year for each dollar spent on PT. However, the PT capacity max sets the maximum proportion of individuals who will adopt PT as their primary mode of transportation for commuting purposes. For instance, if the PT capacity max is set at 0.6, it means that a maximum of 60 per cent of all adopters (i.e., AV, CV, and PT) are PT adopters.

This study highlights the importance of considering various factors when modelling PT demand. The results can be used to inform policy decisions and transportation planning.

#### 3.3.2. Network Capacity Sub-Model

The road network capacity sub-model is illustrated in Figure 4. In this model, the variable ‘road capacity’ represents the total number of cars in the road network that can travel without congestion, and its unit is cars. Level of service is a qualitative measure used to evaluate traffic flow based on factors such as speed, congestion, and density. As a result, the number of vehicles in a given time period in level C condition, “road capacity”, is calculated by multiplying the road length by the level of service C. This sub-model plays a significant role in determining the capacity of the road network and its ability to accommodate the increased use of AVs and CVs. It also assists in identifying potential road congestion and areas where road infrastructure may require upgrades to handle the influx of AVs and CVs. The network capacity sub-model evaluates the combined length of distinct road types within Melbourne. It establishes the overall road capacity, a critical factor influencing the adoption of CVs and AVs, and subsequently impacts the volume of individuals choosing for these vehicle types as the number of trips grows. Table 4 shows the sub-model equations, values, and units for the key variables and stocks.

#### 3.3.3. CV Transitions to AV Sub-Model

Figure 5 shows the CV transitions to the AV sub-model, which presents the transition model between CVs and AVs, where the number of trips generated by each mode depends on the trip cost and time spent. Therefore, if AVs can travel faster and become cheaper and AVs are more attractive than current CVs, people change from public transit (e.g., PT) to auto-based modes [35].

A previous study used two-stage stochastic programming that assumed AV cost and commuting travel time would simultaneously affect AV ownership and adoption rates [36]. Similar to the study conducted by [28], this research assumed that AVs would be available in the market after 2025.

The utility function determines the proportion of people who choose AVs or CVs as their primary mode. ‘AV trip cost’ is expected to decrease over time, represented by the ‘AV trip cost min’ variable and ‘AV trip cost reduction time’ variable. Similarly, ‘AV confidence’ is expected to increase over time with more people adopting AVs and matured technology. ‘AV trip cost’ and ‘AV confidence’ are the two main factors affecting the adoption of AVs compared to the adoption of CVs.

Additionally, the actual VKT will decrease as the ‘car average speed’ decreases due to congestion. The threshold of VKT is determined by the level of service C, called ‘congested VKT per 15 min’, which is calculated based on the total road network capacity (‘Road capacity LOS C’) and the fraction of frequently used road networks during the AM peak (please refer to Section 3.3.2). In contrast, the ‘car desired VKT per 15 min’ variable represents the actual VKT, including both CVs and AVs, which affects the ‘car average speed’. The ‘car average speed’ and ‘Car desired VKT per 15 min’ then influence the ‘CV travel time’ and ‘AV travel time’, ultimately affecting the proportion of individuals choosing these modes (‘CV/AV utility function’). Further, the ‘AV confidence influence rate’ refers to the proportion of individuals positively influenced to choose AVs. This is because those interested in owning AVs tend to rely on their friends for information and recommendations [37].

Therefore, the variables in the transition model are interconnected, and the changes in one variable will affect the other variables, affecting users’ mode choices. Table 5 shows the sub-model equations, values, and units for the key variables and stocks.

### 3.4. Testing

Different tests build confidence for stock-and-flow models [38]. To ensure the reliability and validity of the model, we conducted a series of tests, as recommended by [38], including a model structure test, behavioural test, and boundary test. The model structure test assessed the parameters, boundaries, and overall structural adequacy of the model. For instance, in structure assessment, all the parameters align with the actual system, such as increasing the AV trip cost could make less people choose the AV mode. Similarly, for the boundary assessment, the stock-and-flow model behaviour is sensitive to the removal of existing endogenous elements but insensitive to adding new endogenous elements.

The behavioural test examined the model’s ability to capture and simulate the behaviour of the transportation system realistically. For example, we have changed the value of a single parameter (e.g., PT investment rate) in extreme conditions. Then, the model performs realistically, as the impacted variable (e.g., PT adopters) is within range.

Finally, the boundary test evaluated the sensitivity of the model to changes in the input parameters and boundaries. By passing these tests in the present study, the researchers were confident in the model’s ability to represent the transportation system and evaluate different policy scenarios realistically.

**Figure 6:**
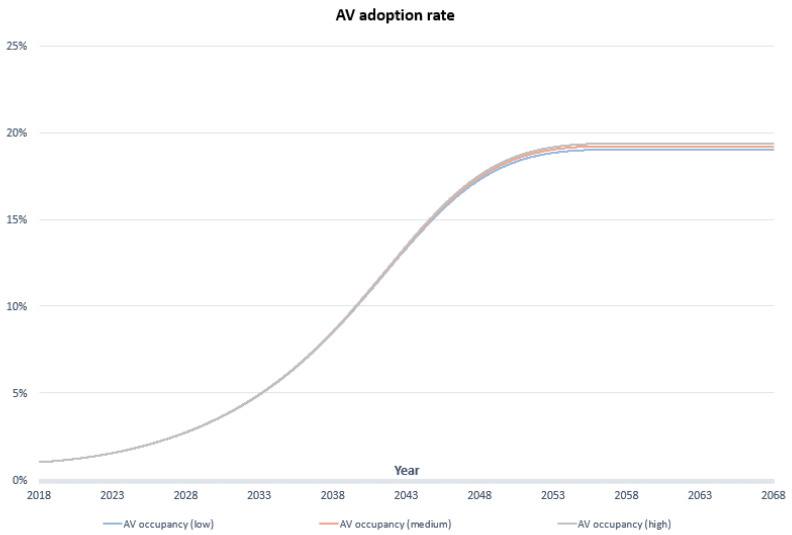
Sensitivity test of AV occupancy.

**Figure 7:**
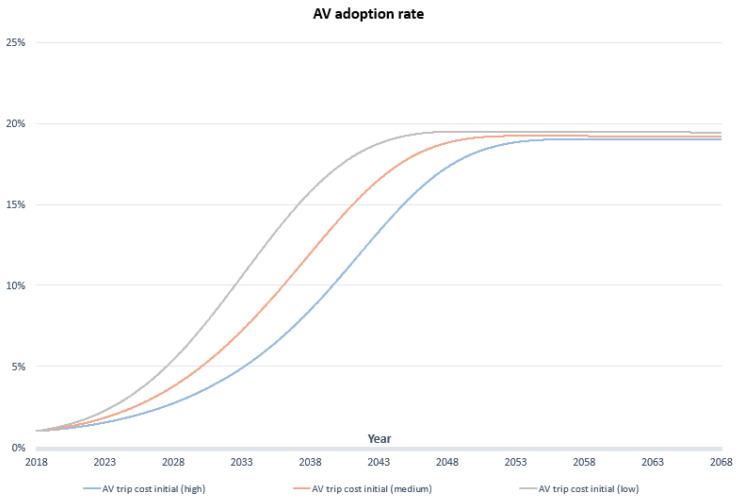
Sensitivity test of AV initial trip cost.

Figure 6 and Figure 7 illustrate the sensitivity analysis conducted on AV occupancy and AV initial trip cost. In Figure 6, different scenarios for AV occupancy—low (average 1.1 person per AV car), medium (average 1.3 person per AV car), and high (average 1.5 person per AV car)—were evaluated. Interestingly, there was minimal variation in AV adoption rates across these scenarios, suggesting that AV occupancy has a minor influence compared to factors like cost, travel time, and social influence.

In Figure 7, the sensitivity test explored AV initial cost through high (800), medium (700), and low (600) scenarios in comparison to the CV trip cost (400). The high-cost scenario exhibited a slow growth in AV adoption rates from 2018 to 2048 due to fewer individuals embracing AVs at a higher cost. However, after 2048, all scenarios converged to the same AV adoption rate, aligning with the decreasing cost trend. Consequently, these sensitivity tests regarding “AV occupancy” and “AV trip cost initial” enhance the model’s credibility and reinforce its validity.

### 3.5. Scenarios

Table 6 outlines the scenarios tested in the model and their respective assumptions. In the base scenario, the maximum fraction of AV adopters remained at 90 per cent, while PT capacity was assumed to be at a 50 per cent fraction level, and the minimum AV trip cost was set at 400. Setting the maximum fraction of AV adopters at 90% is a practical choice, considering that not everyone may fully switch to AVs due to personal preferences or concerns about new technology. As technology improves and people become more confident, a significant portion of the population is expected to embrace AVs. So, in practice, we assume 90% AV adoption instead of 100%. Choosing 50% for PT capacity makes sense because many big cities with well-used public transportation, like New York and London, hover around this utilisation level. Thus, we assume Melbourne’s PT capacity to be 50%, given the city’s current PT usage being below 30%. Additionally, we set AV trip cost min equal to CV trip cost (400) to ensure AV trips remain affordable and competitive, aligning with the cost of conventional trips.

Over time, the model predicted that more CV adopters would transition to AV adopters as trust in AV technology increases and the cost of AVs decreases due to technological advancement. The model also predicted that as network congestion increases, the percentage of trips taken via PT would increase. Other scenarios tested included varying the AV trip cost, PT investment rate, and PT capacity max assumptions to evaluate their effects on mode choice and travel behaviour. These scenarios provide insight into potential future outcomes and the effects of different policy and technological interventions on the adoption of AVs and travel behaviour in Melbourne.

Scenario 1 in the model included the base case with a neutral assumption of 60 per cent for ‘AV adopters max’ because approximately 60 per cent of participants surveyed who had heard of AVs held a positive view of them [32]. The high scenario assumed that ‘AV adopters max’ would be 100 per cent, as it was believed that 100 per cent of individuals could adopt AVs in the next 50 years. In contrast, the low scenario assumed that only 40 per cent of individuals would adopt AVs [32].

In scenario 2, the base case for ‘AV trip cost min’ for calculating the utility function was assumed to be 400 (baseline scenario), the same as the ‘CV trip cost’. However, the Australia-wide survey results showed that around 20 per cent of respondents preferred shared AVs for daily work, reducing the average cost of an AV trip [32]. Thus, the ‘AV trip cost min’ for the low scenario was set to 360. Conversely, for the high scenario, the Australia-wide survey results revealed that the respondents thought AVs were worth more than CVs [32]. As a result, the ‘AV trip cost min’ for the high scenario was assumed to be 430. It was essential to consider preferences and perceptions towards AVs in determining the AV trip cost, as these play a significant role in influencing the adoption rate of AVs. Cost factor was one of the critical factors affecting the adoption rate of AVs. Thus, it was necessary to examine various scenarios to identify the potential effects of AV trip costs on the adoption rate of AVs.

Scenario 3 examined the effect of PT capacity on the transportation system. The assumption for the base case (same as the baseline scenario) was that 50 per cent of the adopters would choose PT as their primary mode, and the PT capacity max was set at 50 per cent. For the low scenario, the PT capacity max was decreased to 30 per cent because currently, PT trips only account for 30 per cent of total trips during the morning peak in Melbourne. This is due to research finding that individuals who already rely on PT as their primary mode of transportation are more inclined to continue using it in the future [39].

The high scenario assumed that 60 per cent of trips were generated by PT in Melbourne for the 24 h period from VITM 2018, and the PT capacity max was set at 60 per cent during the AM morning peak. These assumptions reflect the potential for increased PT usage and the need to ensure that PT capacity can meet growing demand.

The lower and upper scenarios in the study represent the minimum and maximum possible scenarios for AV adopters based on three variables: AV adopters max, AV trip cost min, and PT capacity max. These scenarios help to explore these variables’ potential effects (boundary) on the adoption of AVs and the use of PT.

Table 7 presents the various scenarios for road expansion rates. The low scenario denotes an annual growth of 1 per cent in terms of road network capacity; whereas, the high scenario denotes a growth rate of 3 per cent.

## 4. Results

In this section, we discuss the outcomes under three subsections: baseline scenario, other scenarios, and road expansion and awareness program scenarios.

### 4.1. Baseline Scenario

**Figure 8:**
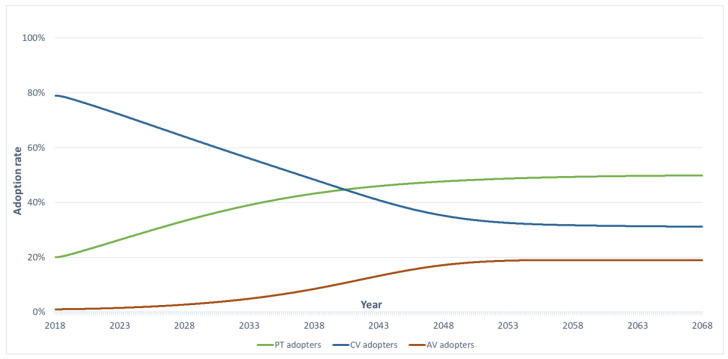
Baseline scenario of AVs, CVs, and PT.

Using the data presented in Table 3, Table 4 and Table 5 and the baseline scenario in Table 6, Figure 8 displays the fluctuation of adoption rates among AVs, CVs, and PT within the 50-year simulation period. In the base year 2018, CV trips accounted for 78 per cent of total trips, while PT trips accounted for 22 per cent. After the trips stabilised in year 30, CV and AV trips by people accounted for 31 per cent and 19 per cent, respectively. The fraction of PT trips remained the same (50 per cent) after year 40, slightly increasing from year 0 (20 per cent). This indicates that after year 30, CV, AV, and PT trips reach equilibrium, and their adoption rates remain stable.

Interestingly, the adoption rates of CVs and PT decrease over time while the adoption rate of AVs increases. This could be due to the technological advancement (lower cost) of AVs and their increased acceptance by the public. As AVs become more affordable and reliable, individuals may switch from CVs to AVs. Similarly, with increasing road network congestion, individuals may shift from CVs and AVs to PT, resulting in a slight increase in PT trips per person until year 40.

Overall, the findings suggest that AVs will gradually replace CVs as another primary mode of transportation in Melbourne. However, PT will still play a significant role in the transportation system, accounting for 50 per cent of total trips by people after year 40. It is noteworthy that alterations in the “PT capacity max” parameter (currently set at 0.5) will impact the proportion of individuals opting for public transport after convergence. Furthermore, while results can fluctuate due to modifications in road capacity, i.e., the utility function and costs of PT, AVs/CVs, confidence gains, and other factors, the overall trend of these travel choices remains consistent. Figure 8 suggests that while the adoption of AVs is projected to increase over time, PT remains pivotal within the transportation network. Consequently, AVs could be effectively integrated as a solution for first- and last-mile connectivity, contributing to the overall efficiency of the transportation system.

### 4.2. Other Scenarios

**Figure 9:**
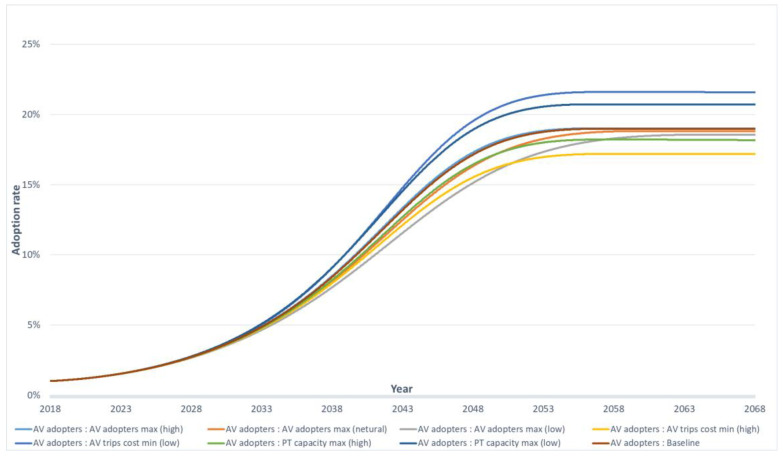
AV adopters in different scenarios.

Figure 9 presents the fraction of AV adopters for 10 scenarios from Table 6, including baseline, lower, and upper cases. Due to road network capacity constraints, the graph shows around 16 to 24 per cent of AV adopters among the 10 scenarios, with the lowest adoption rate from the lower case and the highest from the upper case. The adoption rate of AVs started to increase around year 8, due to the assumption that people would start accepting AVs from 2026 (base year 2018), and stabilised around year 34 (2052). Except for the lower and upper scenarios, the lowest adoption rate (17 per cent) was in the scenario ‘AV trip cost min (high)’, while the highest adoption rate (22 per cent) was in the scenario ‘AV trip cost min (low)’. These results show that the cost of AV trips is an important factor in determining the adoption rate of AVs.

Compared with the scenario ‘AV trips cost min(high)’ and ‘PT capacity max(high)’, the AV adopters rate of scenario ‘AV adopters max(low)’ exceeded these two scenarios after year 32. This indirectly proves that the cost of AV trips is the most important factor compared with ‘AV adopters max’ and ‘PT capacity max’. The second- and third-highest AV adopters rate scenarios were ‘PT capacity max(low)’ and ‘AV adopter max(high)’, meaning PT demand might reach a high level due to lower capacity, resulting in more people switching to AVs. Therefore, it is essential to consider the balance between PT capacity and AV adoption rate to ensure a smooth transition to AVs. The evaluation of these 10 scenarios, featuring different values for “AV adopters max”, “AV trip cost min”, and “PT capacity max”, reveals a spectrum of adoption rates spanning from 16% to 24%. This implies that these variables introduce relatively minor uncertainties in the results. These findings indicate that cost, whether through financial incentives or subsidies, emerges as the primary determinant influencing the number of individuals who opt for AV adoption once the adoption rate stabilises. These findings can be helpful for policymakers and stakeholders to make informed decisions regarding AV adoption policies and strategies.

### 4.3. Road Expansion and Awareness Program Scenarios

**Figure 10:**
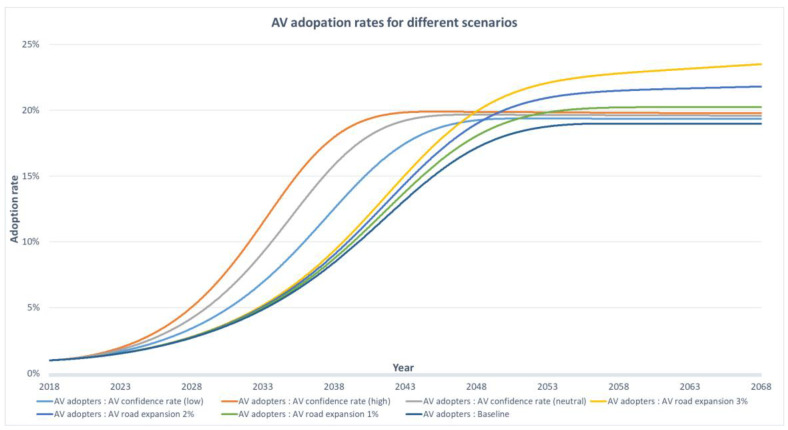
AV adopters in different road expansion and awareness program scenarios.

Figure 10 shows the adoption rate of AVs under different road expansion and awareness program scenarios. For the road expansion program, the baseline scenario assumed a 1 per cent annual growth rate in road network capacity, while the high scenario assumed a 3 per cent growth rate. The baseline scenario showed an AV adoption rate of 19 per cent, while the high scenario showed a rate of approximately 24 per cent that continued slightly even after year 50. This suggests that a 1 per cent increase in road network capacity could lead to a 2 per cent increase in the AV adoption rate. However, higher road expansion rates can result in a longer time for AV adoption to stabilise due to increased space on the road. Interestingly, there is little difference in AV adoption rates between road expansion scenarios in the first 20 years. This could be because road network capacity is not a significant determining factor, and cost remains the most critical factor influencing AV adoption.

Further, the study suggests that awareness programs could be more effective than road investment programs in increasing AV adoption rates, particularly between years 4 and 24. However, even with the low and high scenarios for the influence rate, the AV adopter rate remained lower than the low road expansion scenario, at 19.3 per cent and 19.7 per cent, respectively. Therefore, although awareness programs could lead to a more rapid increase in adoption rates, road investment programs are more likely to result in higher adoption rates in the long term.

## 5. Discussion

In this section, we discuss the following three aspects: AV adoption, awareness program, and cost.

### 5.1. AV Adoption

A study conducted by [40] developed a framework to forecast the adoption of AVs in Nashville, US. The study projected that AVs would likely capture a 50 per cent market share by year 18 and an 80 per cent market share by year 31, which differs from our study’s findings (10 per cent market share by year 18 and 23 per cent after year 30). This variance may be attributed to the difference in the transportation culture between Australia and the US. Unlike Australia, the US has a car-centric culture, which may lead to a more rapid adoption of AVs. Similarly, [41] proposed a simulation-based framework using a multinomial logit model to predict Americans’ adoption of CAVs under different scenarios. The authors found that privately owned AVs would be 24.8 per cent in year 30, compared with 18 per cent in the present study, and this result was based on an annual 5 per cent price decrease and the same willingness to pay value.

Additionally, a dynamic approach for designing AV subsidies to accelerate the early deployment of AVs was developed by [42]. The present study also highlights the importance of cost as a critical factor for adoption, which can be addressed through optimal subsidies. Reference [43] used a discrete choice model by incorporating it into the dynamic model with AV subsidies and infrastructure investment as inputs. That study concluded that the optimal subsidy increased from USD 10,000 in year 1 to USD 20,000 in year 60, when AV market penetration was 50 per cent.

In contrast to our study, [44] employed agent-based modelling and considered the reduced value of time of AVs caused by parking restrictions and increased congestion. The study concluded that AVs would decrease transit ridership by 75 per cent, which differs from the present findings that showed an increase in public transit ridership due to congestion and reduced AV costs. In a similar study, [23] used SD modelling to discover that traffic volume would considerably increase, leading to higher congestion equilibrium levels and more VKT. It is, therefore, imperative for the present study to consider the induced traffic volume, as we assumed a relatively stable total number of trips.

Automated on-demand mobility services, such as Uber and taxis, could potentially see a reduction in PT trips by 9–10 per cent in Singapore during peak hours with the introduction of AVs alongside private vehicles [45]. This is because some individuals may shift from PT to AVs due to lower costs compared with existing taxis and on-demand mobility services.

This study’s findings suggest that policymakers and stakeholders need to consider the effect of congestion levels on AV adoption rates when developing policies to promote AVs. It is essential to address current congestion levels, as this can influence the adoption rate of AVs. Therefore, road expansion and awareness programs could be a more effective approach to promoting AV adoption. Further, cost is the most important factor in determining the adoption rate of AVs, and optimal subsidies could be used to make AVs more affordable and competitive in the market.

### 5.2. Awareness Programs

According to [46], social influence and public acceptance are two crucial factors necessary for the widespread adoption of AVs. To encourage the adoption of AVs, governments should work with manufacturers to promote their usefulness and create favourable conditions that foster social influence and public acceptance.

Ref. [47] used SD modelling to find that a lack of customer acceptance was the main barrier to AV adoption. The authors suggested that awareness programs can address this issue, which can help increase the adoption rate. As suggested by [48], the societal dimension of AVs as part of governance processes is important for the transition from CVs to AVs. These recommendations from past studies are consistent with the present study’s finding that an awareness program could increase AV adoption rates more quickly than a road investment program.

Although awareness programs can drive an initial surge in AV adoption rates (e.g., from 2028 to 2048), this study indicates that their effect may diminish over time. Therefore, policymakers should consider longer-term strategies, such as investment in AV infrastructure (e.g., charging stations), especially when future cars become electric AVs, to sustain and increase adoption rate.

### 5.3. Cost

In this study, cost was identified as the most significant factor affecting adoption rates. Similarly, in a study conducted by [49], a lab experiment was carried out in a mixed traffic environment consisting of AVs and CVs to explore the mode preferences of individuals. Participants who received complete information about mode and cost considered perceived cost and inertia during the decision-making process. According to [50], various trade-offs, such as travel time cost, waiting time cost, miles travelled, and operational cost, were captured by considering AVs in private and shared mobility systems. The researchers concluded that technological advancement is necessary to promote AV adoption due to the lower cost of AVs, which aligns with this study’s findings.

Additionally, [42] found that optimal subsidies can serve as both an incentive for AV manufacturers to innovate and improve their products and a means of providing competitive pricing to attract potential consumers. Moreover, the research conducted by [51] showed that individuals tended to be more responsive to the cost of the vehicle and the provision of exclusive lanes, which is consistent with the findings of the present study regarding AV trip costs and road expansion programs. The cost factor could also significantly influence individuals’ decisions to adopt AVs in Ireland [52].

Therefore, policymakers could consider providing optimal subsidies to AV manufacturers to innovate and improve their products and offer competitive pricing to attract potential consumers. Further, governments could invest in AV infrastructure, such as exclusive lanes, to provide a more seamless and efficient travel experience for AV users. Policymakers should also consider the balance between PT capacity and AV adoption rates to ensure a smooth transition to AVs, which could lead to a reduction in private car usage and, consequently, contribute to reducing greenhouse gas emissions.

## 6. Conclusions

This study used an SD modelling approach to investigate the effect of AVs on the mode shift between CVs, AVs, and PT in Melbourne, Australia, by systematically considering a range of factors, such as road network, vehicle cost, PT supply, and congestion level. The study also highlights the importance of cost as a critical factor in adoption, which can be addressed through optimal subsidies. Further, the adoption rate of AVs was found to be affected by road network capacity and awareness programs. While higher road expansion rates could result in a longer time for AV adoption rates to stabilise, awareness programs could lead to a more rapid increase in adoption rates. However, road investment programs are more likely to result in higher adoption rates in the long term. Therefore, it is important to facilitate the transition from CVs to AVs in a seamless manner so that road network can accommodate both types of vehicles during the transition period.

The increasing prevalence of AVs in Australia may have significant implications for mobility patterns, particularly in ride-hailing services [53]. This could result in a more congested network, as future travel demand is expected to be primarily carried out via private AVs, with most passengers using shared patterns [6]. Therefore, it is crucial to investigate the adoption rates of AVs in various potential scenarios, including those involving ride-hailing services. Understanding the potential effect of AVs on ride-hailing services will help policymakers develop strategies to manage traffic congestion and ensure a sustainable transportation system.

The SD model developed in this study has the potential to assist planners, policymakers, and researchers to evaluate the potential effect of AVs on the transportation system and plan accordingly to minimise adverse effects and maximise the benefits of AVs. Likewise, analogous to the approach described in reference [54], the adaptation of monitoring strategies in response to evolving conditions holds applicability in making well-informed decisions regarding mode shifts within dynamic transportation systems. However, this study has several limitations that need to be addressed in future research. First, the study only considered a single metropolitan area and assumed that AVs would be available to everyone equally. In reality, AV adoption rates may vary across regions due to factors such as urban design, travel patterns, and demographic characteristics. Thus, future studies should explore the adoption rates of AVs in different regions and the factors that influence them.

The study made three assumptions regarding AV adoption rates in Melbourne, Australia, including a constant 2 per cent growth rate in total trips over time. However, in reality, AVs may induce demand for travel by providing more convenient and accessible transportation, increasing total trips. Thus, future research should explore the potential induced demand due to AV adoption. Additionally, applying equilibrium constraints to traffic assignment models could enhance the model’s accuracy by characterising route choice behaviour and vehicle preference, as suggested in the literature [36].

## Figures and Tables

**Table 1:** A list of abbreviations used in this study.

Abbreviation	Explanation
AVs	Automated Vehicles
CVs	Conventional Vehicles
PT	Public Transport
LoS	Level of Service
CAVs	Connected and Autonomous Vehicles
VITM	Victorian Integrated Transport Model
EVs	Electric Vehicles
SD	System Dynamic
VISTA	Victorian Integrated Survey of Travel and Activity
EVs	Electric Vehicles
DTP	Department of Transport and Planning
CBD	Central Business District
VKT	Vehicle Kilometre Travelled

**Table 2:** System Dynamic Approaches Review.

Purpose	Variable	Strength	Conclusion	Future Study Suggestion/Limitation
To evaluate the construction scale of urban rail for traffic, economy, and society [26]	GDP, population, accident, gas emission, congestion degree; construction scale was a policy variable	Presented the effect of the urban rail system on urban traffic, economy, society, and environment; guided transportation infrastructure planning	As the mileage of urban rail increased, the number of cars increased; appropriate construction of urban rail would help	Some variables need more research, such as sociology, economics, and demography
To evaluate the effects of AVs on mode choice and broader transportation system [23]	Travel time, public transit fare, traffic volume, adequacy of PT, etc.	Three different scenarios to investigate the effect on mode choice and mobility	Better to obtain public acceptance of AVs as shared-use vehicles or PT tools before establishing the mindset of private vehicles	Public discussion should be initiated to fully understand views on AVs when AVs are in the market
To evaluate the innovation diffusion of AVs in the long term [24]	Technology maturity, research and development funds, attractiveness, purchase price, and fleet	Complex and dynamic innovation systems of AVs and six levels of AVs were represented	System was highly uncertain due to different market penetration levels and policies adopted	Further research could focus on gaining more knowledge of factors affecting the diffusion of AVs by leveraging this model
To evaluate the mobility effects of AVs [25]	Mode choice, travel time, and time of day choice	Uncertainties were incorporated into penetration rates, capacity, and value of time	AVs could cause increased car trips and level of congestion	Extend the model by considering travel time reliability, road pricing policy, and ride-sharing
A useful approach for optimising individuals’ mobility and guiding city planners [16]	Rail, car, bus, and air customers (mode choice)	What factors influence people’s choices and can model their behaviours; several scenarios were included (sensitive to price, trip duration, and need to stay overnight)	Customers were not sensitive to price, trip duration, need to stay overnight, or need to use additional means of transport	Future research should be parametrised to identify more details for individual platforms
Adoption of EVs [17]	Economic utility (cost, infrastructure convenience, and vehicle technology) and social utility	Complex interaction and how feedback can affect EV adoption	Consumers’ vague perceptions and pilot of EV projects led to delays in EV adoption; however, social commerce helped	Future research should focus on EV adoption through combinations of incentive plans
To evaluate the effects of AV adoption on greenhouse gas emissions [27]	Emissions, fleet, and adoption	Life cycle assessment to assess the various scenarios in the medium to long term	To decrease greenhouse gas emissions, the government should manage vehicle travel speeds, provide subsidies, and increase the renewable electricity supply	Further research needs to focus on developing the model in conjunction with other methods to support the investigation of greenhouse emission process

**Table 4:** Network capacity sub-model’s parameters, equations, and values.

Parameter Name	Unit	Value (Equation)	Source/Explanation
Local collector density	Car/km	11.2	LoS C standard (HCM 2016)
Local collector length	km	5572.5	VITM provided by DTP
Secondary arterial density/ Rural unsealed density/Ramp terminal density/Primary divided density/Primary undivided density/CBD density	Car/km	13.7	LoS C standard
Secondary arterial length	km	3626.84	VITM provided by DTP
Rural unsealed length	km	741.2	VITM provided by DTP
Level crossing length	km	84.83	VITM provided by DTP
Ramp terminal length	km	29.58	VITM provided by DTP
Freeway density	Car/km	16.2	LoS C standard (HCM 2016)
Freeway length	km	2707.51	VITM provided by DTP
Primary divided length	km	4113.7	VITM provided by DTP
Primary undivided length	km	4010.57	VITM provided by DTP
CBD length	km	64.04	Sourced from the VITM model to provide input for this analysis, signifying the road length within Melbourne’s central business district (CBD) in kilometres
CBD density	Car/km	13.7	Acquired from the traffic engineering standard, specifically the level of service C standard, to ascertain the optimal traffic density for vehicle movement to travel smoothly

Notes: DTP (Department of Transport and Planning); VISTA (Victorian Integrated Survey of Travel and Activity).

**Table 5:** CV transitions to AV sub-model’s parameters, equations, and values.

Parameter Name	Unit	Value (Equation)	Source/Explanation
AV/CV desired VKT per 15 min	Car × km	AV/CV trips per 15 min × Car average speed LoS C × “15 min”	Maximum car capacity in the network that does not lead to congestion
AV/CV occupancy	Person/Car	1.1	Average number of persons per car
AV/CV trips per 15 min	Car	AV/CV trips per 15 min per person/AV/CV occupancy	Number of AV/CV trips for any 15 min during AM peak
AV/CV trips per 15 min per person	Person	Passenger trips per 15 min × AV/CV adopters	Number of AV/CV trips among total trips generated by private vehicle trips and PT trips
AV/CV fleet travel time	Car × hour	AV/CV desired VKT per 15 min/Car average speed	Vehicle × km/km/h equals vehicle × h
AV/CV travel time	Minute	AV fleet travel time × min per h/AV trips per 15 min	Average AV/CV travel time per vehicle
AV/CV utility function	N/A	−1.55–0.066 × AV/CV travel time − 0.004 × AV/CV trip cost	It is an AV/CV utility function to determine the probability of choosing AV/CV mode
Car average speed	km/hour	Car average speed LoS C − (Car desired VKT per 15 min − Congested VKT per 15 min)× (Car average speed LoS C − Car average speed gridlock)/(Gridlock VKT per 15 min − Congested VKT per 15 min)	Vehicle speed decreases as VKT exceeds the congestion threshold
Car average speed LoS C	km/hour	48.1	VITM provided by DTP
Congested VKT per 15 min	Car × km	Road capacity LoS C × Road use fraction × Car average speed LoS C × “15 min”	Threshold for congestion in a network level depends on average vehicle speed (travel in a smooth way) and road capacity
Road use fraction	N/A	0.62	This is the assumed value as there are some roads that are seldomly used in Victorian network
AV adopters initial	Dmnl	0.01	This must be greater than zero to avoid a ‘floating point error’ due to division by zero in ‘AV travel time’ at t = 0

Notes: AV (automated vehicle); CV (conventional vehicle); DTP (Department of Transport and Planning); VITM (Victorian Integrated Transport Model); and VKT (vehicle kilometres travelled).

**Table 6:** Various scenarios implemented in the model.

Scenario	Parameter Name	Unit	Value		
			Low	Neutral	High
Baseline	AV adopters max	N/A			90%
	AV trip cost min	N/A		400	
	PT capacity max	N/A		50%	
1a	AV adopters max	Fraction	40%		
1b				60%	
1c					100%
2a	AV trip cost min	N/A	360		
2b		N/A			430
3a	PT capacity	Fraction	30%		
3b					60%
Lower	AV adopters max	Fraction	40%		
	AV trip cost min	N/A			430
	PT capacity max	Fraction			60%
Upper	AV adopters max	Fraction			100%
	AV trip cost min	N/A	360		
	PT capacity max	Fraction	30%		

Notes: AV, automated vehicle; na, not applicable; and PT, public transport.

**Table 7:** Road expansion and awareness program implemented in the model.

Scenario	Parameter Name	Unit	Value			
			Baseline	Low	Neutral	High
Road expansion program	Road expansion rate	Fraction	0%			
				1%		
					2%	
						3%
AV awareness program	AV confidence influence rate	Fraction	40%			
				60%		
					80%	
						100%

Notes: AV, automated vehicle.

## Data Availability

Not applicable.
